# Implication of G Protein-Coupled Receptor 43 in Intestinal Inflammation: A Mini-Review

**DOI:** 10.3389/fimmu.2018.01434

**Published:** 2018-06-22

**Authors:** Guan Yang, Siyuan Chen, Baichuan Deng, Chengquan Tan, Jinping Deng, Guoqiang Zhu, Yulong Yin, Wenkai Ren

**Affiliations:** ^1^Guangdong Provincial Key Laboratory of Animal Nutrition Control, Subtropical Institute of Animal Nutrition and Feed, College of Animal Science, South China Agricultural University, Guangzhou, Guangdong, China; ^2^Jiangsu Co-Innovation Center for Important Animal Infectious Diseases and Zoonoses, Joint International Research Laboratory of Agriculture and Agri-Product Safety of Ministry of Education of China, College of Veterinary Medicine, Yangzhou University, Yangzhou, China

**Keywords:** G protein-coupled receptor 43, inflammation, short chain fatty acids, inflammatory bowel diseases, intestinal microbiota

## Abstract

Short chain fatty acids (SCFAs, e.g., acetate, propionate, and butyrate) are a subset of fatty acids that are produced by gut microbiota during the fermentation of dietary fiber. They modulate different processes in the gastrointestinal tract and play various positive roles in mediating the intestinal health. Most beneficial roles of SCFAs in the gastrointestinal tract are mediated by directly activating its receptor, G protein-coupled receptor 43 (GPR43, also known as FFAR2). Various recent studies have demonstrated the role of GPR43 in intestinal inflammatory diseases, such as inflammatory bowel diseases. These SCFAs-mediated regulations of intestinal health are associated with neutrophil chemotaxis, T cell differentiation, activation, and subsequent cytokines production. Therefore, GPR43 could potentially be a drug target for intestinal inflammatory diseases. In this review, we review the current knowledge on the regulatory mechanisms associated with GPR43 in intestinal inflammation. The role of GPR43-mediated regulation of antibody responses is also discussed.

## Introduction

Inflammatory bowel diseases (IBD) are chronic inflammatory diseases with unknown etiology. It comprises the chronic relapsing inflammatory disorders: Crohn’s disease and ulcerative colitis (1). Although the pathogenesis of IBD remains unknown, it is widely accepted that the pathogenesis of IBD involves immune and environmental factors, including intestinal inflammation and gut microbiota (1–6). Next Generation Sequencing and Genome-Wide Association Studies have revealed strong associations of polymorphisms in some gene loci with IBD (7). Recent evidence also showed the single amino acid change would directly affect the protein function and conformation, suggesting the possible role of single amino acid polymorphisms in genetic predisposing of IBD (8). Inflammation is a cascade of responses triggered by immune cells and cytokines to defend the body against injuries, harmful stimuli, infection, or microbial toxins (9). As the intestinal mucosa is in constant contact with a diverse array of exogenous microorganisms and substances, inflammatory responses are necessary for the maintenance of gastrointestinal homeostasis (1, 10). However, sustained and excessive inflammation responses in the intestinal mucosa are associated with various gastrointestinal pathologies, such as IBD (9, 11). Notably, numerous investigations have indicated that the homeostasis of the intestinal microbiota could positively influence intestinal immunity, and protect against the development of intestinal inflammatory diseases, while intestinal dysbiosis leads to intestinal diseases (12, 13). For example, a feature of human IBD is a change in the number of “healthy” microbiota such as *Bifidobacterium* and *Bacteriodes* (14). The mechanisms involved in these processes are not fully unraveled, but a group of molecules known to be involved in the cross talk between the microbiome and the gut immune system are short chain fatty acids (SCFAs). SCFAs refer to carboxylic acids with aliphatic tails of less than six carbons such as formic, acetic, propionic, butyric, isobutyric, valeric, isovaleric, and 2-methyl-butyric acids that are produced by gut microbiota during the fermentation of unabsorbed carbohydrates and dietary fiber. SCFAs are found in high concentrations in the intestinal tract, ranging from 70 to 140 mM in proximal colon and 20 to 70 mM in the distal colon, with acetate as the predominant SCFA (15). In addition to providing energy to gut epithelium and peripheral tissues, SCFAs also promote intestinal epithelial integrity and aid in the repair of wounded epithelium (16). They also involved in many physiological processes in humans and rodents by acting as signaling molecules. Intriguingly, various studies have demonstrated the beneficial effects of fermentable dietary fiber or SCFAs in the progression of colitis, reducing risks of cardiovascular disease, colon cancer, obesity, and diabetes (17–21). These SCFA-mediated regulations are achieved through the activation of G protein-coupled receptors (GPCRs), inhibition of histone deacetylase (HDAC), stimulation of histone acetyltransferase activity, and stabilization of the hypoxia-inducible factor (22–25). GPCRs can be classified into the category of moonlighting proteins (26–28) as they can carry out different types of functions. Details on the effects of SCFAs and GPCRs on IBD have been reviewed by others (29). In this review, we summarize current knowledge on the regulatory mechanisms associated with G protein-coupled receptor 43 (GPR43)-mediated effects in intestinal inflammation.

## The SCFA Receptor GPR43

The superfamily of GPCRs is one of the largest families of proteins in the mammalian genome and shares a conserved structure composed of seven transmembrane helices (30). SCFA-sensing GPCRs include GPR41 (FFAR3), GPR43 (FFAR2), GPR109, and olfactory factor 78, which are present in intestinal epithelial cells, adipocytes, and immune cells (31). Most notable among the SCFA targets is the GPR43, which is abundantly expressed in the intestines, adipose tissues, with the highest expression found in immune cells such as monocytes and neutrophils (32–35), suggesting the possible regulatory functions of GPR43 in the recruitment of these cell types during inflammatory responses *in vivo*. GPR43 has drawn much attention in recent years, and might be the mechanism through which SCFAs directly regulate the immune cells and the process of inflammatory diseases (36, 37). It could be activated by SCFAs, with acetate and propionate serve as the most potent activators followed by butyrate and other SCFAs (32, 33). Activation of GPR43 by acetate and propionate, in turn, induce both Gα_i_ and Gα_q_, which inhibits cAMP production but increases intracellular levels of calcium ions (32–34). Many studies have investigated the role of GPR43 in regulating intestinal inflammatory responses. GPR43-deficient mice have exacerbated disease symptoms in models of dextran sulfate sodium (DSS)-induced colitis as evidenced by reduced colon length, an increased disease activity index, severe inflammation, and increased myeloperoxidase (MPO) activity in the colon, and these symptoms could not be ameliorated by 150 mM (37) or 200 mM (36) of acetate treatment, although same doses of acetate treatment significantly improved the disease course of DSS-induced colitis in WT mice (36, 37). These results suggested that GPR43 mediates the protective effects of SCFA in intestinal inflammation. However, contradictory roles of GPR43 were reported. Sina et al. reported that GPR43-deficient mice have reduced tissue damage and inflammatory cell infiltration in acute DSS colitis (4% DSS in water for 6 days) (38). GPR43-deficient mice were even found to be protected from chronic colitis (e.g., reduced MPO, increased colon length) induced by three cycles of 2% DSS administration (one cycle: 5 days 2% DSS in drinking and 5 days regular water) (38). A possible explanation for the different outcomes for these studies may be due to differences in the mice. As Maslowski et al. (36) and Masui et al. (37) used mice provided by Deltagen, whereas those used by Sina et al. (38) were made by Lexicon Pharmaceuticals. In addition, Kespohl et al. found that 100 mM sodium butyrate treatment for 3 weeks exacerbated disease severity in 1.5% DSS model of germ free mice possibly through inducing the expression of T-bet and IFN-γ in a GPR43-independent manner (39). A recent study in comparing the effects of SCFA and GPR43 agonists on intestinal barrier function found that SCFA could enhance the intestinal barrier function and inhibit the immune cell activation but in a GPR43-independent pathway (16). Furthermore, although GPR43 plays positive roles in mounting the acute inflammatory responses after 2,4,6-trinitrobenzene sulfonic acid (TNBS) treatment, reduced acute immune responses in the GPR43-deficient mice results in the delayed clearance of pathogens at early time points (40). Taken together, these results indicate the importance of GPR43 in regulating gut immunity as well as in linking diet, gut microflora, and the body’s inflammatory responses. These results also suggest different interpretations of experimental outcomes are likely and largely dependent on the experimental models and time points chosen for analysis.

## GPR43 and Neutrophil Chemotaxis

Polymorphonuclear leukocytes (PMNs; neutrophils) are the most abundant form of white blood cells, comprising approximately 60% of all white blood cells during non-infectious states in humans. It is well known that neutrophils play a key role in the battle against invading pathogens by delivering the anti-microbial molecules and reactive oxygen intermediates (41). In addition, neutrophils also help to regulate other immune responses by generating signals that recruit monocytes and dendritic cells, and help to determine macrophage polarization (42). Thus, efficient trafficking of neutrophils to the infected sites in the body is critical for host defense against pathogenic challenge. The key pathological feature of both human and experimental colitis models is the significant migration of PMNs into the lamina propria and epithelial layer (43, 44), which could be regulated by many factors such as the presence of various chemokines and upregulation of integrins (45, 46). Among all these factors that regulate the migration of PMNs, SCFAs–GPR43 axis attracted a lot of interest.

Previous study has demonstrated the role of sodium acetate and sodium propionate in inducing the chemotaxis of human PMNs with a bell-shaped dose–response curve with an optimal concentration of 1 mM (33). In rodents, SCFAs could stimulate the neutrophil migration, which is associated with an increased production of the chemoattractant cytokine CINC-2αβ and the increased expression of L-selectin on the neutrophil surface (47). In addition, SCFAs induced activation of GPR43 is implicated in directional migration of neutrophils *in vitro*. GPR43-dependent signaling is able to phosphorylate p38 mitogen-activated protein kinase (MAPK) and activate MAPK pathway, which has been considered as a major determinant contributor to chemotaxis in PMNs (48), indicating the important role of SCFA-induced GPR43-dependent signaling on neutrophil migration. In another *in vitro* study using bone marrow-derived neutrophils from wild-type and GPR43-deficient mouse, activation of the GPR43 in wild-type but not GPR43-deficient neutrophils support neutrophil motility and direction sensing, which allows neutrophils to efficiently migrate toward a source of acetate, propionate, or butyrate (49). Mechanistically, this GPR43-dependent chemotaxis requires PI3Kγ, Rac2, p38, and ERK (49).

GPR43-deficient mice develop exacerbated inflammation in models for colitis likely contributed to higher production of inflammatory mediators and increased neutrophil infiltration/activation (36). GPR43-deficient neutrophils showed increased chemotaxis toward bacterial products (fMLP) and to the complement fragment C5a (36) or the classic chemoattractant, CXCL1 (50). These data further confirm GPR43 as a chemotactic receptor for neutrophils. However, despite the fact that GPR43 regulating the recruitment of the neutrophils, its relevance with chemokine production and expression of adhesion molecules, which may be relevant to the effect of these fatty acids on leukocyte recruitment, is still not clear. The interaction of SCFA–GPR43 on neutrophils may play an immune modulation role at the intestinal level, inhibiting acute bacterial transmigration, but also an anti-inflammatory role in chronic inflammatory responses.

## GPR43 and Cytokines Expression

Naïve CD4^+^ T cells differentiate into a variety of subsets including T helper (Th)1, Th2, and Th17 cells as defined by their pattern of cytokine production and immune function (51, 52). Cytokines in the intestinal epithelium are important components initiated by the immune system for maintaining normal gut homeostasis. A disturbance of the cytokines profile in favor of inflammation may lead to the disease state, such as the observation in IBD. SCFAs can induce the generation of Tregs and affect Th1, Th2, and Th17 cell differentiation and activation [reviewed in Ref. (53)]. Studies have also shown that SCFAs modulate colitis by regulating inflammatory cytokines production in a GPR43-dependent pathway (37, 40). However, the roles of GPR43 in the production of inflammatory cytokines are controversial. Masui and colleagues showed that DSS-treated GPR43-deficient mice exhibited severer disease symptoms with higher IL-17 and TNF-α but similar IL-10 expression compared with wild-type mice (37). Treatment of wild-type mice with 150 mM acetate markedly improved the disease indexes associated with decreased IL-17 and TNF-α and increased IL-10 level, while no effects observed in GPR43-deficient mice in model of DSS-induced colitis (37). Blocking the GPR43 signaling by anti-GPR43 antibody also inhibited the effect of acetate suggesting this regulation of cytokine production is GPR43-dependent (37). In a different colitis model induced by administration of ethanol or TNBS, Kim et al. found that GPR43-deficient mice showed reduced inflammatory responses and had slower immune responses against *Citrobacter rodentium* infection (40). Failure to clear *C. rodentium* at early time points was associated with low frequencies of Th1 and Th17 cells in the colon as well as delayed induction of inflammatory cytokines, IFN-γ, and chemokines (CXCL1 and CXCL2). The different roles of GPR43 in these two studies are likely caused by the different induction of colitis. DSS colitis switches from a Th1–Th17-mediated acute inflammation to a Th2-mediated inflammatory response as disease becomes chronic while TNBS colitis exhibits heightened Th1–Th17 response in the chronic state (54). In agreement with the results by Masui et al., they also found that GPR43 was required to recruit leukocytes and activate effector T cells in the intestine (40). Furthermore, the induced production of chemokines and cytokines were associated with the activation of ERK1/2 and p38 MAPK signaling pathways (40). In addition, a recent study demonstrated that acetate promotes T-cell differentiation into Th1 and Th17 cells (55). Interestingly, they found this effect of SCFAs on T cell differentiation is independent of GPR41 or GPR43, but dependent on direct HDAC inhibitor activity (55), suggesting that GPR43 may not be directly involved in SCFA regulation of naïve T cell differentiation. However, this result is in direct conflict with another study showing that SCFAs directly suppress HDACs in a GPR43-dependent manner (56). Butyrate also reduces surface expression of chemoattractant receptors C5aR and CXCR2 in neutrophils through GPR43 receptor (36). Hence, more research on the biology of GPR43 is required to confirm the role of GPR43 in regulating inflammation in different models.

## Concluding Remarks

**Figure 1:**
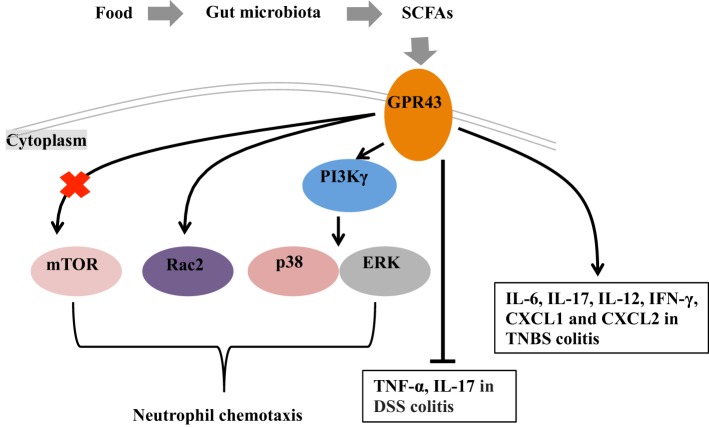
Mechanisms of short chain fatty acids (SCFAs)-mediated intestinal health. Under fed conditions, SCFAs are produced in the gut by fermentation of the fiber by the gut microbiota. G protein-coupled receptor 43 (GPR43), which serve as the receptor of SCFAs, plays a role in the intestinal inflammation through regulating the neutrophil chemotaxis and mediating the cytokine expression. The mechanisms for GPR43 to regulate the neutrophil chemotaxis are associated with the activation of mTOR, Rac2, p38, and ERK. In the absence of GPR43, IL-17 and TNF-α are highly produced in dextran sulfate sodium (DSS)-induced colitis model and lead severe colitis symptoms. GPR43 is also important for the induction of inflammatory cytokines (i.e., IL-6, IL-17, IL-12, and IFN-γ) and chemokines (CXCL1 and CXCL2) in TNBS-induced colitis, and further promote the clearance of *Citrobacter rodentium* infection in this model.

SCFA levels decreased remarkably in IBD patients compared with healthy individuals supporting the notion that SCFAs play an important role in the pathogenesis of IBD (57, 58). Administration of SCFAs or prebiotics that promote the SCFA production has long been used to treat IBD patients (59–63). In addition to their role in regulating intestinal T cell differentiation in gut mucosa, SCFAs can also affect the gut B cell development and antibody responses. Although there is a variety of evidence indicating that the GPR43 is involved in intestinal inflammation, whether GPR43 is a positive or negative modulator in intestinal inflammation remains controversial. This controversial also apply to the SCFA’s regulation on B cells. Kim et al. reported that SCFAs promote plasma B cell differentiation by controlling the gene expression that is necessary for antibody production in an SCFA receptor-independent manner (64). However, Wu et al. showed that acetate promotes IgA production with GPR43-dependent manner (65). Taken together, GPR43 participates in the regulation of intestinal inflammation may be associated with neutrophil recruitment and cytokine production (Figure 1). Future studies are expected to reveal the regulatory mechanisms associated with GPR43 in intestinal inflammation. The discovery of drugs acting on GPCRs has been extremely successful, thus GPR43 may also represent a promising therapeutic target for the treatment of intestinal diseases, such as IBD.

## Author Contributions

GY and WR have designed the review article and approved the final manuscript. GY wrote the review article. BD and CT helped to find references. SC, JD, GZ, and YY revised the review article.

## Conflict of Interest Statement

The authors declare that the research was conducted in the absence of any commercial or financial relationships that could be construed as a potential conflict of interest.
